# The Story of #GetMePPE and GetUsPPE.org to Mobilize Health Care Response to COVID-19 : Rapidly Deploying Digital Tools for Better Health Care

**DOI:** 10.2196/20469

**Published:** 2020-07-20

**Authors:** Shuhan He, Ayotomiwa Ojo, Adam L Beckman, Suhas Gondi, Megan Ranney, Marian Betz, Jeremy S Faust, Esther Choo, Dara Kass, Ali S Raja

**Affiliations:** 1 Center for Innovation in Digital HealthCare Massachusetts General Hospital Boston, MA United States; 2 Department of Emergency Medicine Massachusetts General Hospital Boston, MA United States; 3 Harvard Medical School Boston, MA United States; 4 Department of Emergency Medicine Warren Alpert Medical School Brown University Providence, RI United States; 5 Department of Emergency Medicine School of Medicine University of Colorado Denver, CO United States; 6 Department of Emergency Medicine Brigham and Women’s Hospital Department of Emergency Medicine Boston, MA United States; 7 Department of Emergency Medicine Oregon Health and Science University Portland, OR United States; 8 Department of Emergency Medicine Vagelos College of Physicians and Surgeons Columbia University New York, NY United States

**Keywords:** digital health, getusppe, getmeppe, COVID-19, personal protective equipment, protection, Twitter, pandemic, health care worker

## Abstract

Physicians, nurses, and other health care providers initiated the #GetMePPE movement on Twitter to spread awareness of the shortage of personal protective equipment (PPE) during the coronavirus disease (COVID-19) pandemic. Dwindling supplies, such as face masks, gowns and goggles, and inadequate production to meet increasing demand have placed health care workers and patients at risk. The momentum of the #GetMePPE Twitter hashtag resulted in the creation of a petition to urge public officials to address the PPE shortage through increased funding and production. Simultaneously, the GetUsPPE.org website was launched through the collaboration of physicians and software engineers to develop a digital platform for the donation, request, and distribution of multi-modal sources of PPE. GetUsPPE.org and #GetMePPE were merged in an attempt to combine public engagement and advocacy on social media with the coordination of PPE donation and distribution. Within 10 days, over 1800 hospitals and PPE suppliers were registered in a database that enabled the rapid coordination and distribution of scarce and in-demand materials. One month after its launch, the organization had distributed hundreds of thousands of PPE items and had built a database of over 6000 PPE requesters. The call for action on social media and the rapid development of this digital tool created a productive channel for the public to contribute to the health care response to COVID-19 in meaningful ways. #GetMePPE and GetUsPPE.org were able to mobilize individuals and organizations outside of the health care system to address the unmet needs of the medical community. The success of GetUsPPE.org demonstrates the potential of digital tools as a platform for larger health care institutions to rapidly address urgent issues in health care. In this paper, we outline this process and discuss key factors determining success.

## Introduction

**Figure 1:**
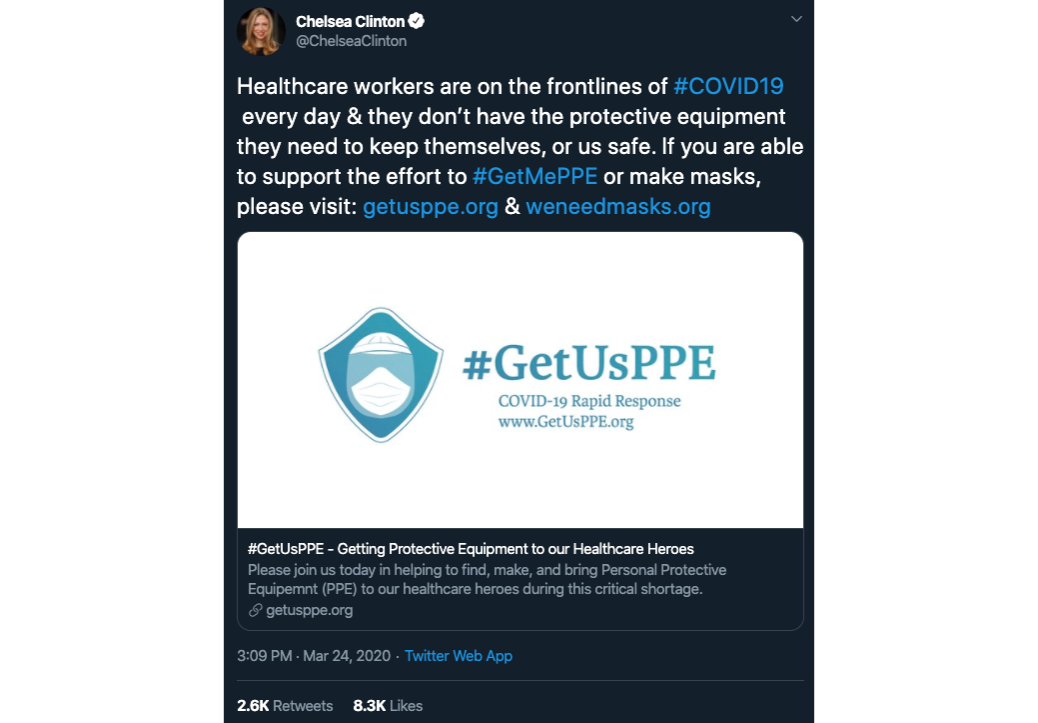
Example tweet from an individual who increased the viewership of #GetMePPE [4].

On March 17, 2020, the Twitter hashtag #GetMePPE went viral [1-3] as thousands of nurses, physicians, and other health care providers posted pictures of themselves with personal protective equipment (PPE). The pictures were accompanied by attestations of dwindling—or in some cases completely absent—stocks of face masks, N95 respirators, and other supplies, even as providers began to evaluate and treat patients with coronavirus disease (COVID-19). The hashtag drew an outpouring of public support and media attention [4] amplifying the PPE shortage (Figure 1); a website, GetMePPE.org, obtained over 62,000 signatures for its online petition for increased government support of, and funding for, PPE [5].

 Simultaneously, a separate group launched GetUsPPE.org [6] with the goal of connecting people who needed PPE with individuals or organizations who had some available. The two groups came together on March 21, with the GetUsPPE.org webpage taking on the #GetMePPE petition as well as the coordination of PPE donation delivery. Only 10 days later, the site had over 120,000 views, the petition had over 10,000 signatures, and over 1800 hospitals and PPE suppliers had entered their information into the continuously updated database. Currently, one month after the merger, a web application with automated attribution and a last-mile system has facilitated the delivery of hundreds of thousands of items of PPE in three trial cities (getusppe.org/data), and partnerships have been formed with the American Medical Association, the American Hospital Association, and a number of specialty societies [7]. The GetUsPPE.org website was rapidly launched to collect available supply and demand requests. Textbox 1 shows a list of the types of facilities that can request PPE on the website.

**Types of health care facility designations for the Request Donations page on the GetUsPPE.org website.**

- Acute care hospital
- Freestanding emergency room
- Field hospital
- Hospital overflow facility
- Emergency medical services
- Urgent care clinic
- Nonacute care hospital
- Inpatient psychiatric facility
- Inpatient rehabilitation facility
- Residential substance treatment facility
- Nursing home or skilled nursing facility
- Assisted living facility or group home
- Homeless shelter
- Correctional facility or detention center
- Hospice
- Dialysis center
- Outpatient clinic (primary care or specialist)
- Public health clinic or community health center
- Indian or Tribal healthcare facility (any kind)
- Outpatient rehabilitation facility
- Dental or oral surgery clinic (providing urgent/emergency care)
- Home health agency
- COVID-19 test site
- Ophthalmic provider
- Clinical laboratory
- Pharmacy
- Mortuary or coroner's office
- Social services organization
- Other

Much like just-in-time manufacturing [8], we propose that this type of rapid website deployment will be a solution in the future when urgent health care scenarios arise.

## Needs

At the time of writing, 56/60 US jurisdictions (93.3%) have reported cases of COVID-19 infection [9]; most states are experiencing sustained community spread. While analysis of US statistics is ongoing, in analyses from Wuhan, China, 26% of patients received ICU care, and the mortality rate was 4.3% [10]. PPE is critical to protect health care workers at the front lines of caring for these patients. According to US Centers for Disease Control guidelines for use of PPE in US hospitals [11], both N-95 filtering facemask respirators and powered air-purifying respirators should be accompanied by the use of a full-face shield, helmet, or headpiece. Prior experience with the Ebola virus and severe acute respiratory syndrome (SARS) affirm that PPE provides “the last physical barrier” between health care workers and infectious body fluids [12,13].

Despite this, health care providers across the United States are lacking basic PPE, including gowns and masks, as they care for sick patients with COVID-19 [14]. Evidence from past epidemics suggests that clinical workers are at much higher risk than the general population of being infected [15,16]. PPE shortages not only compromise the safety of health care workers but also put patients at risk. As health care providers fall sick, fewer of us are available to care for patients. The lack of PPE also results in higher infection rates within hospitals as staff move from patient to patient without proper protection. In the Wuhan case analysis, hospital-related COVID-19 transmission occurred in 41% of patients [10].

## Application of Digital Tools

In less than 12 hours over the course of a weekend, a group of medical students, doctors, software engineers, and volunteers from around the US built and launched a web-based platform, GetUsPPE.org, to enable the public to answer the call for help. On the website, organizations requesting PPE report their name, organization type, and address, followed by their PPE needs (such as PPE types and conditions of acceptance). People or organizations interested in donating PPE provide their contact details and information about their supplies, and they indicate if they are available to deliver or ship them. We were immediately flooded with heartwarming support and offers to help, not only from a variety of existing sources of PPE but also from novel additive manufacturing facilities and homemade makers. More importantly, the site enabled us to collect signatures to push for government action, process PPE donations from a public now keenly aware of the problem, and facilitate distribution of the PPE to health care workers in need. The website now supports global affiliates, including India as a prominent partner [17].

We attribute the success of our digital platform to three key components. First, there was unity around a clear and common cause. Importantly, the effort was not limited to physicians but was extended to all staff impacted by the shortage of PPE. Through firsthand stories of paramedics facing exposure in the field, nurses treating patients who were coughing in their faces, housekeeping staff cleaning rooms without adequate protection, pharmacists assisting with intensive care unit medication dosing without masks, and concerned friends and family, it was clear that our platform was needed for health care workers of all types.

Second, as health care providers in the “trenches,” we leveraged social media to share what we were witnessing and give all stakeholders a voice. Using the #GetUsPPE hashtag, physicians shared photographs of themselves without proper PPE, video tutorials teaching each other how to create homemade PPE [18], and images of store shelves fully stocked with boxes of N95 masks [19] while hospitals had none [20]. Stories circulated quickly on Twitter and on other social media platforms, including stories of emergency physicians hospitalized after contracting COVID-19 on the job [21]. Later, the stories became more uplifting, with updates of community members coming together to support health care workers and donate PPE to hospitals and other health care organizations.

Finally, the digital platform itself is intuitive and easy for users to negotiate, and it was not difficult to develop. The website, while initially primitive, was created literally overnight with the aid of the increasingly large number of tools that facilitate building and maintaining websites for the uninitiated; after its quick launch, the site experienced rapid growth. The live launch and traffic catalyzed further development and collaboration with subject matter experts to quickly iterate multimodal technological solutions, including databases (eg, to store information regarding which hospitals are most in need of PPE), matching algorithms (eg, to match PPE donations with the closest hospital in need), and logistics capabilities (eg, to coordinate the manufacturing of PPE and delivery to hospitals).

## Lessons

Our experience with #GetMePPE and GetUsPPE.org exemplifies the untapped potential of digital platforms to mobilize and enable a ready and willing public to alleviate some of the largest barriers faced by health care workers. The COVID-19 pandemic has illuminated the deficiencies of our health care supply chain, rendering traditional sources of support inadequate. Fortunately, digital literacy is increasing rapidly worldwide, and the general public is increasingly embracing digital media. However, the medical field has yet to take full advantage of this opportunity.

Social media is just one example of a digital opportunity for health care providers to help increase the quality and efficiency of health care [22]. Current use of social media by physicians is mostly one-way rather than interactive [23]. However, it is critical for health care providers and organizations to create and participate in websites. While social media is essential for raising public awareness and excitement, a website enables health care providers to action this momentum to manifest tangible benefits for themselves and their patients.

## Conclusion

GetUsPPE.org is just one example of the successful use of digital tools not only to voice needs, such as those of people working on the front lines of a pandemic, but to mobilize available resources at a speed we have rarely seen before. This initiative has demonstrated the efficacy, reach, and speed of digital platforms; however, it by no means represents the potential the medical community could achieve with full support and buy-in from larger national organizations or the federal government. To date, we have taken a bottom-up, grassroots approach, operating in entrepreneurial fashion, often donating our personal time and resources. It is interesting to imagine what we could accomplish with formal support. In this time of unprecedented crisis and need, we health care professionals have a responsibility to do everything we can to offer the best possible care, including ensuring that we can continue serving our patients without sacrificing either our safety or theirs.

## Abbreviations

COVID-19: coronavirus disease
PPE: personal protective equipment
SARS: severe acute respiratory syndrome

## References

[1] Gondi S, Beckman AL, Deveau N, Raja AS, Ranney ML, Popkin R, He S (2020). Personal protective equipment needs in the USA during the COVID-19 pandemic. Lancet.

[2] Glenza J (2020). The Guardian.

[3] (2020). @nytimes.

[4] (2020). @ChelseaClinton.

[5] GetUsPPE.

[6] GetUsPPE.

[7] GetUsPPE.

[8] Tack P, Victor J, Gemmel P, Annemans L (2016). 3D-printing techniques in a medical setting: a systematic literature review. Biomed Eng Online.

[9] US Centers for Disease Control and Prevention.

[10] Wang D, Hu B, Hu C, Zhu F, Liu X, Zhang J, Wang B, Xiang H, Cheng Z, Xiong Y, Zhao Y, Li Y, Wang X, Peng Z (2020). Clinical Characteristics of 138 Hospitalized Patients With 2019 Novel Coronavirus-Infected Pneumonia in Wuhan, China. JAMA.

[11] (2019). US Centers for Disease Control and Prevention.

[12] Fischer WA, Weber D, Wohl DA (2015). Personal Protective Equipment: Protecting Health Care Providers in an Ebola Outbreak. Clin Ther.

[13] Weber DJ, Rutala WA, Schaffner W (2010). Lessons learned: Protection of healthcare workers from infectious disease risks. Crit Care Med.

[14] Ranney ML, Griffeth V, Jha AK (2020). Critical Supply Shortages - The Need for Ventilators and Personal Protective Equipment during the Covid-19 Pandemic. N Engl J Med.

[15] Chan-Yeung M (2004). Severe acute respiratory syndrome (SARS) and healthcare workers. Int J Occup Environ Health.

[16] Beckman AL, Gondi S, Forman HP (2020). Health Affairs.

[17] GetUsPPE.

[18] (2020). @mystifide.

[19] (2020). @drdanchoi.

[20] (2020). @choo_ek.

[21] (2020). American College of Emergency Physicians.

[22] McGowan BS, Wasko M, Vartabedian BS, Miller RS, Freiherr DD, Abdolrasulnia M (2012). Understanding the factors that influence the adoption and meaningful use of social media by physicians to share medical information. J Med Internet Res.

[23] Campbell L, Evans Y, Pumper M, Moreno MA (2016). Social media use by physicians: a qualitative study of the new frontier of medicine. BMC Med Inform Decis Mak.

