# Correction: The Story of #GetMePPE and GetUsPPE.org to Mobilize Health Care Response to COVID-19 : Rapidly Deploying Digital Tools for Better Health Care

**DOI:** 10.2196/46028

**Published:** 2023-01-31

**Authors:** Shuhan He, Ayotomiwa Ojo, Adam L Beckman, Suhas Gondi, Megan Ranney, Marian Betz, Jeremy S Faust, Esther Choo, Dara Kass, Ali S Raja

**Affiliations:** 1 Center for Innovation in Digital HealthCare Massachusetts General Hospital Boston, MA United States; 2 Department of Emergency Medicine Massachusetts General Hospital Boston, MA United States; 3 Harvard Medical School Boston, MA United States; 4 Department of Emergency Medicine Warren Alpert Medical School Brown University Providence, RI United States; 5 Department of Emergency Medicine School of Medicine University of Colorado Denver, CO United States; 6 Department of Emergency Medicine Brigham and Women’s Hospital Department of Emergency Medicine Boston, MA United States; 7 Department of Emergency Medicine Oregon Health and Science University Portland, OR United States; 8 Department of Emergency Medicine Vagelos College of Physicians and Surgeons Columbia University New York, NY United States

In “The Story of #GetMePPE and GetUsPPE.org to Mobilize Health Care Response to COVID-19 : Rapidly Deploying Digital Tools for Better Health Care” (J Med Internet Res 2020;22(7):e20469), one error was noted.

Due to a system error, the name of one author, Megan Ranney, was replaced with the name of another author on the paper, Suhas Gondi. In the originally published paper, the order of authors was listed as follows:

Shuhan He, Ayotomiwa Ojo, Adam L Beckman, Suhas Gondi, Suhas Gondi, Marian Betz, Jeremy S Faust, Esther Choo, Dara Kass, Ali S Raja

This has been corrected to:

Shuhan He, Ayotomiwa Ojo, Adam L Beckman, Suhas Gondi, Megan Ranney, Marian Betz, Jeremy S Faust, Esther Choo, Dara Kass, Ali S Raja

In the originally published paper, the ORCID of author Megan Ranney was incorrectly published as follows:

0000-0003-3048-3472

This has been corrected to:

0000-0002-8450-9642

In the originally published paper, Megan Ranney's affiliation was incorrectly published as follows:

Harvard Medical School, Boston, MA, US

This has been corrected to:

Department of Emergency Medicine, Warren Alpert Medical School, Brown University, Providence, RI, United States

The correction will appear in the online version of the paper on the JMIR Publications website on January 31, 2023, together with the publication of this correction notice. Because this was made after submission to PubMed, PubMed Central, and other full-text repositories, the corrected article has also been resubmitted to those repositories.

